# The Influence of Metabolic Syndrome on Potential Aging Biomarkers in Participants with Metabolic Syndrome Compared to Healthy Controls

**DOI:** 10.3390/biomedicines12010242

**Published:** 2024-01-22

**Authors:** Drahomira Holmannova, Pavel Borsky, Ctirad Andrys, Jan Kremlacek, Zdenek Fiala, Helena Parova, Vit Rehacek, Monika Esterkova, Gabriela Poctova, Tereza Maresova, Lenka Borska

**Affiliations:** 1 Institute of Preventive Medicine, Faculty of Medicine in Hradec Kralove, Charles University, 500 03 Hradec Kralove, Czech Republicborka@lfhk.cuni.cz (L.B.); 2 Institute of Clinical Immunology and Allergology, University Hospital Hradec Kralove and Faculty of Medicine in Hradec Kralove, Charles University, 500 03 Hradec Kralove, Czech Republic; 3 Institute of Medical Biophysics, Faculty of Medicine in Hradec Kralove, Charles University, 500 03 Hradec Kralove, Czech Republic; 4 Institute of Clinical Biochemistry and Diagnostics, University Hospital Hradec Kralove and Faculty of Medicine in Hradec Kralove, Charles University, 500 03 Hradec Kralove, Czech Republic; 5 Transfusion Department, University Hospital Hradec Kralove, 500 03 Hradec Kralove, Czech Republic

**Keywords:** aging, metabolic syndrome, NLRP3, klotho, telomerase, AGEs, DNA/RNA damage GDF11/15, NAD^+^, sirtuin 1, follistatin, vitamin D

## Abstract

Background: Biological aging is a physiological process that can be altered by various factors. The presence of a chronic metabolic disease can accelerate aging and increase the risk of further chronic diseases. The aim of the study was to determine whether the presence of metabolic syndrome (MetS) affects levels of markers that are associated with, among other things, aging. Material and Methods: A total of 169 subjects (58 with MetS, and 111 without metabolic syndrome, i.e., non-MetS) participated in the study. Levels of telomerase, GDF11/15, sirtuin 1, follistatin, NLRP3, AGEs, klotho, DNA/RNA damage, NAD^+^, vitamin D, and blood lipids were assessed from blood samples using specific enzyme-linked immunosorbent assay (ELISA) kits. Results: Telomerase (*p* < 0.01), DNA/RNA damage (*p* < 0.006) and GDF15 (*p* < 0.02) were higher in MetS group compared to non-MetS group. Only vitamin D levels were higher in the non-MetS group (*p* < 0.0002). Differences between MetS and non-MetS persons were also detected in groups divided according to age: in under 35-year-olds and those aged 35–50 years. Conclusions: Our results show that people with MetS compared to those without MetS have higher levels of some of the measured markers of biological aging. Thus, the presence of MetS may accelerate biological aging, which may be associated with an increased risk of chronic comorbidities that accompany MetS (cardiovascular, inflammatory, autoimmune, neurodegenerative, metabolic, or cancer diseases) and risk of premature death from all causes.

## 1. Introduction

Aging is a physiological process associated with progressive structural and functional degeneration and an increased incidence of chronic age-related diseases (cardiovascular and neurodegenerative diseases, diabetes, cancer, altered immune functions, etc.) and all-cause mortality [1]. Biological age, as opposed to chronological age, can be influenced by many stimuli, exposure to physical and psychological stress, chemicals, infections, excessive fat intake, obesity, etc. [2,3]. Biological age and its progression can be monitored by markers of aging, which are associated with changes in the genome (telomere length, genomic instability, telomerase activity, epigenetic changes), mitochondrial function, cellular senescence, stem cell exhaustion, loss of proteostasis, dysregulation of nutrient sensing, disruption of intercellular communication, etc. [4,5,6].

Metabolic syndrome is a complex disorder associated with increased body weight, central obesity, elevated blood pressure, dyslipidemia, hyperinsulinemia and insulin resistance, low-grade chronic low-grade inflammation, increased oxidative stress, and the risk of cardiovascular, neurodegenerative, and autoimmune diseases, as well as cancer, nonalcoholic fatty liver disease, diabetes, etc. [6,7,8]. The presence of metabolic syndrome and/or its components is associated with accelerated biological aging and premature death [8,9].

The individual components of metabolic syndrome do not usually occur in isolation, and one can trigger the onset or worsening of the other. For example, hyperinsulinemia is thought to be one of the drivers of aging. It is associated with inflammation, insulin resistance, increased lipogenesis, and fat storage. Hyperinsulinemia is a risk factor for obesity and cardiovascular disease, including high blood pressure [10]. In the study, we focus on the metabolic syndrome, which may include all of these factors. The aging process in the presence of metabolic syndrome is thus a reflection of all the components involved in its pathogenesis.

In our study, we selected markers that reflect aging and/or metabolic syndrome, biochemical parameters (blood lipids), telomerase, GDF11 and 15 (Growth Differentiation Factor 15 and 11), sirtuin 1, follistatin, NLRP3 (NOD-like Receptor family Pyrin domain-containing 3), AGEs (Advanced Glycation End products), klotho, DNA/RNA damage, NAD^+^ (Nicotamide Adenine Dinucleotide), and vitamin D.

According to studies, life expectancy is also related to telomere length and the length of telomeres shortens with aging. The enzyme telomerase maintains its length and genomic stability and thus prevents cellular senescence and aging [11]. Therefore, a decrease in telomerase expression and function may be associated with accelerated aging. On the other hand, increased activity is associated with tumorigenesis and other pathological processes accompanied by increased cellular division [12,13]. 

Like telomerase, sirtuin 1 also affects genome stability. Sirtuin is closely related to NAD^+^. Sirtuin 1 is a NAD^+^-dependent protein deacetylase, which is involved in DNA repair processes [14]. Sirtuin 1 has anti-aging and anti-inflammatory effects, but its expression is suppressed by inflammation and regulates glucose and lipid metabolism [15,16,17]. NAD^+^ is not only important for the sirtuin 1 function but also regulates numerous cellular functions, metabolic pathways and metabolic homeostasis, DNA repair, senescence, immune cell activity, etc. NAD^+^ levels decrease during aging [18].

Aging is also accompanied by an increase in the levels of AGEs and advanced glycation end products. AGEs are generated mainly by the nonenzymatic glycation of lipids and proteins. They can originate in the organism or be ingested as food. Their levels increase during aging and can trigger inflammatory reactions and the production of reactive oxygen species (ROS) [19,20].

The higher production of ROS (reactive oxygen species) can be induced not only by the presence of AGEs but also by inflammation and mitochondrial dysfunction. Oxidative stress is closely linked to DNA/RNA damage and the formation of oxidized DNA/RNA bases (e.g., 8-hydroxyguanosine). Adduct formation is associated with altered replication and DNA regeneration [21,22]. Increased ROS production is also one of the factors that activate NLRP3. 

NLRP3 is a multiprotein complex, an inflammasome, which process inactive IL-1β and IL-18 into an active proinflammatory form. The activation of NLRP3 is associated with acute and chronic inflammation, metabolic disorders, and aging. Its blockade alleviates inflammation and slows down biological aging [23,24,25].

Oxidative stress, inflammation, and other processes associated with aging can alter the expression of GDF11 and GDF15. Although both growth factors belong to the same family, the transforming growth factor family, their effects are completely different. GFD11 is considered a rejuvenating factor which can prolong lifespan. The increased levels of GDF15 reflect the presence of cellular stress, tissue damage, and an increased risk of chronic disease and death [26,27,28]. On the other hand, GDF15 suppresses food intake and may help to treat metabolic diseases [29].

Another parameter we measured was klotho. Klotho is known as an anti-aging factor that regulates a variety of pathways involving aging, including insulin and Wnt signaling. It also inhibits inflammation and reduces oxidative stress. The decline in klotho levels accelerates aging [30,31,32].

A very interesting factor in relation to aging and metabolic disorders is follistatin. Follistatin is an extracellular protein expressed in almost all tissues that antagonizes several members of the transforming growth factor beta family, myostatin, and activins. It is associated with metabolic diseases, modulates adipose tissue metabolic function, and promotes the browning of adipose tissue. It also regulates glycemia and insulin resistance, β cell survival, muscle growth, inhibits activin-induced inflammation and fibrotic processes, etc. [33,34,35].

The last parameter selected was vitamin D. Although vitamin D is classified as a vitamin, it is more a hormone in nature and function. Its deficiency is associated with a wide variety of chronic diseases (cardiovascular, neurodegenerative, and inflammatory diseases, dyslipidemia, diabetes, metabolic syndrome, obesity, etc.) [36,37,38].

In the previous study, we found that aging parameters were correlated with BMI and biochemical parameters (triglycerides, cholesterol) that are associated with metabolic syndrome. This study aimed to determine whether the presence of metabolic syndrome affects the values of markers associated with biological aging. 

## 2. Materials and Methods

The presented study builds on a study by Borsky et el., which was conducted with the same group of healthy individuals. For the assessment of the influence of metabolic syndrome, previous and new results were used and evaluated. To ensure the completeness of methods in this article, the previously used methods are also described [39].

A total of 169 volunteers were included in our study. Participants were divided into groups according to the presence of metabolic syndrome, namely MetS (58) and no MetS (111), and by age and presence of metabolic syndrome: MetS and non-Mets (without metabolic syndrome) aged under 35 years, 35–50 years, and over 50 years.

Exclusion criteria included: the presence of any inflammatory disease, pregnancy, or anti-inflammatory, non-steroidal, or other chronic medication. All participants signed an informed consent form before the start of the study. The study was carried out in accordance with the Declaration of Helsinki, and the protocol was approved by the Ethics Committee of the Faculty Hospital in Hradec Kralove, Czech Republic.

### 2.1. Metabolic Syndrome Diagnostics

Metabolic syndrome was diagnosed according to the National Cholesterol Education Program Adult Treatment Panel (NCE/ATPIII) criteria. To be diagnosed with metabolic syndrome, at least three of the five criteria were required.

- Waist circumference ≥ 102 cm for men and ≥88 cm for women;
- Fasting glucose levels ≥ 5.6 mmol/L or known treatment for diabetes;
- Triglyceride level (TAG) ≥ 1.7 mmol/L;
- High-density lipoproteins levels (HDL cholesterol) < 1.03 mmol/L for men and <1.30 mmol/L for women;
- Blood pressure ≥ 130 mmHg/≥85 mmHg.

### 2.2. Biochemical Parameters

Biochemical parameters were analyzed from the vein blood at the Institute of Clinical Biochemistry and Diagnostics (University Hospital and Faculty of Medicine in Hradec Kralove, Charles University). These parameters include fasting glucose levels, TAG, total cholesterol, LDL, HDL, nonHDL, progesterone, estrogen, testosterone, and DHEA (dehydroepiandrosterone).

### 2.3. Blood Sampling

Blood samples were collected from the cubital vein into BD Vacutainer tubes at the Transfusion Centre of the University Hospital Hradec Králové. The blood samples were processed, and the blood serum obtained by centrifugation was frozen and stored at −70 °C until analysis. Repeated thawing and freezing were avoided.

### 2.4. Biomarker Detection

Selected biomarkers were measured with commercial ELISA kits according to the manufacturer’s instructions and the absorbance values were read at 450 nm on a Multiskan RC ELISA reader (Thermo Fisher Scientific, Waltham, MA, USA).

- Levels of NLPR3 were evaluated using Human NALP/NLRP3 ELISA Kit (LifeSpan BioSciences, Inc., Seattle, WA, USA); samples were not diluted. The sensitivity was 0.313–40 ng/mL.
- Levels of Klotho were analyzed using Human Klotho ELISA Kit (Cusabio, Cloud-Clone Corp, Katy, TX, USA); samples were not diluted. The detection range was 0.156–10 ng/mL.
- Levels of telomerase were measured with Human Telomerase (TE) ELISA Kit ELISA Kit (Cusabio, Houston, TX, USA); samples were two-fold diluted. The detection range was 0.31–40 ng/mL.
- Levels of AGEs were determined with Human Advanced Glycation End Products (Agens) ELISA Kit (Cusabio, Houston, TX, USA); samples were not diluted. The detection range was 0.78–50 µg/mL.
- DNA/RNA damage was determined using DNA/RNA Oxidative Damage EIA Kit (Cayman Chemical Company, Ann Arbor, MI, USA); samples were 100-fold diluted. The detection range was 10–30,000 pg/mL 8-hydroxy 2-deoxy guanosine.
- Levels of GDF11 were detected by Human GDF11/GDF11 ELISA Kit (LifeSpan BioSciences, Inc., Seattle, WA, USA); samples were not diluted. The detection range of the kit was 7.8–1000 pg/mL.
- Levels of GDF15 were evaluated using Quantikine ELISA Human GDF15 Kit (R&D Systems, Minneapolis, MN, USA); samples were four-fold diluted. The detection range was 93.6–6000 pg/mL.
- Levels of NAD were measured with Enzyme-linked Immunosorbent Assay Kit for Nicotinamide Dinucleotide (NAD) (Cloud-Clone Corp. (Katy, TX, USA)); samples were 20-fold diluted. The detection range was 2400–200,000 ng/mL.
- Levels of Sirtuin-1 were determined using Human SIRT 1/Sirtuin 1 ELISA Kit (LifeSpan BioSciences, Inc., Seattle, WA, USA); samples were 50-fold diluted. The detection range was 3.9–250 ng/mL. Results were converted from pg/mL to ng/mL due to smaller numbers.
- Levels of Follistatin were evaluated using Quantikine ELISA Human Follistatin Kit (R&D Systems, MN, USA); samples were not diluted. The detection range was 125–16,000 pg/mL.
- Levels of Vitamin D were analyzed using 25-OH Vitamin D ELISA Kit (EUROIMMUN, Lubeck, Germany); samples were 26-fold diluted. The detection range was 4–120 ng/mL.

### 2.5. Statistical Analysis

After evaluating the normality of the distribution using the Anderson–Darling test, parametric or nonparametric tests were performed. Correlations between the selected parameters were assessed through either Pearson’s or Spearman’s correlation test. Differences between treatment groups were analyzed using Student’s t-test or Wilcoxon’s rank-sum test. The null hypothesis was rejected if the probability level (*p*) was less than 0.05 (alpha level).

## 3. Results

### 3.1. Demographic Data

In our study, we included 169 participants (85 men and 84 women) that were divided into groups according to the presence of metabolic syndrome: metabolic syndrome (MetS; n = 58) and without metabolic syndrome (non-MetS; n = 111). Participants were also divided according to the age: under 35 (30 females, 28 non-MetS, 2 MetS; 27 males, 20 non-Mets, 7 MetS), 35–50 (28 females, 18 non-MetS, 10 MetS; 21 males, 13 non-MetS, 18 MetS), and over 50 years (26 females, 19 non-MetS, 7 MetS; 27 males, 13 non-MetS, 14 MetS). There were 20 smokers, 21 ex-smokers, and 128 nonsmokers in our study. We did not find any differences in the values of measured markers or correlations between smokers and nonsmokers in any group. 

The values of biochemical markers and parameters associated with MetS were higher in the MetS group compared to non-MetS (Table 1). 

### 3.2. Values of Biomarkers of Aging in Non-MetS and MetS Participants

In subjects under 35, significant differences between non-MetS and MetS were found only in follistatin and vitamin D. In participants in the group 35–50, there were significant differences between non-MeS and MetS in the levels of GDF15 and follistatin. In participants over 50, there were no differences in levels of analyzed markers (Table 2).

### 3.3. Correlations among Selected Parameters

We evaluated correlations between all measured parameters in non-MetS and MetS participants and found several correlations. Importantly, there was a difference in positive and negative correlations between non-MetS and MetS participants (Table 3, Table 4 and Table 5). Klotho, AGE, follistatin, and GDF15 correlated with blood lipids but only in the non-MetS group.

## 4. Discussion

Aging is a natural process that leads to the gradual deterioration of the body, increased susceptibility to disease, and death. While chronological aging cannot be regulated, biological aging depends on a wide range of factors. Biological age does not have to coincide with chronological age and can be determined by markers of aging. These markers reflect the processes that occur during aging (inflammation, altered metabolism, nutrient sensing, telomere shortening, mitochondrial damage, oxidative stress, etc.).

We assessed the levels of selected markers in a healthy population compared to individuals with metabolic syndrome and investigated whether individuals with metabolic syndrome experience accelerated biological aging. We found significant differences between both groups in the values of the selected parameters and in the correlation between parameters (see results and/or Supplementary Materials). This implies that different changes occur in the body of healthy people, which may also be related to biological aging. They can accelerate it, which is especially noticeable in the 35–50-year-old group. In the group aged over 50, the values are comparable and there are no statistical differences.

Almost all parameters accompanied with MetS (BMI, blood pressure, total cholesterol, LDL, nonHDL, triglycerides, fasting glucose), except HDL, were higher in the MetS group compared to the non-MetS group.

Telomerase levels were higher in the MetS group, without subgroup differences. Telomerase negatively correlated with fasting glucose and GDF11, positively correlated with sirtuin 1, DNA/RNA damage, and AGEs. In the non-MetS group, telomerase additionally correlated with NAD^+^.

In some chronic diseases, the activity of telomerase may be higher, and it is activated mainly by cell division, autoimmune diseases, or cancer [40]. Telomerase expression is induced by inflammation, especially via NFκB signaling pathways. It has also been shown that the suppression of TNFα-induced NFκB activity in airway smooth muscle cells occurred after treatment with telomerase activity inhibitors [41]. MetS is associated with low-grade inflammation in which the activation of NFκB plays a crucial role [42]. AGEs and DNA/RNA damage values that positively correlated with telomerase are pro-inflammatory factors. Therefore, higher levels of telomerase were documented in our MetS group.

The positive correlation between telomerase and sirtuin 1 was expected since sirtuin 1 induces telomerase expression [43]. The negative correlation between telomerase and glucose can be explained by the fact that telomerase maintains the replicative capacity of pancreatic beta cells and thus insulin production, which lowers glucose levels. Thus, higher telomerase activity may reduce glycemic values. However, it should be stated that more studies to prove such hypothesis should be performed [44]. Interestingly, the negative correlation between telomerase and GDF11 is inconsistent with known data. Wang et al. found that GDF11 deficiency (in vitro) is associated with telomere shortening and reduced telomerase expression [45]. Similar results are described in the study by Chen et al., who also found that GDF11 enhanced telomerase activity [46]. 

Follistatin is significantly involved in metabolic processes and aging. Gene therapy with the cytomegalovirus vector with the telomerase of follistatin extended the median lifespan by 41.4% and 32.5% in mice. It also improved glucose tolerance, physical performance, and the maintenance of body weight [47].

Although its levels did not significantly differ between the non-MetS and the MetS groups, slightly higher levels were observed in the MetS group. Subgroup analysis showed that follistatin levels were significantly higher in the under-35, non-MetS subgroup, and inversely, levels were higher in the 35–50 MetS subgroup. There were no differences in follistatin levels in the over-50s subgroup. The results suggest that follistatin levels depend on the presence of MetS, as well as age. In the MetS group, follistatin correlated with age, AGEs, and GDF15, in non-MetS group with total cholesterol, nonHDL, TAG, and GDF15. Data related to metabolic syndrome are lacking, so we will use comorbidities associated with metabolic syndrome to discuss our results.

Wu et al. found that elevated levels of follistatin are associated with the risk of type II diabetes (DMII) because it induces adipose tissue insulin resistance [33]. Hansen et al. analyzed blood samples from patients with DMII and healthy controls. The levels of follistatin were increased in patients and correlated (only in patients) with fasting glucose, TAG, and total cholesterol [48]. The results of the correlations are inconsistent with our study in which these correlations were detected in the non-MetS group. Sylow et al. proved the role of follistatin in DMII but did not confirm its elevation in obese persons [34]. We saw slightly higher levels in the MetS group, especially in the 35–50 subgroup, which is consistent with the results of other authors. However, interestingly, an increase in follistatin levels was observed in the non-MetS group aged under 35. A possible explanation could be that young people are more physically active and levels of follistatin can be elevated by exercise [49].

Vitamin D values were higher in non-MetS compared to the MetS group. In the subgroup analysis, vitamin D levels were also higher in the non-MetS and in the under-35 subgroup. In non-MetS, vitamin D negatively correlated with BMI and NLRP3 and positively correlated with HDL, GDF11, and AGEs, while in MetS, it negatively correlated with BMI and waist circumference. Wang et al. showed that lower levels of vitamin D are a risk factor for MetS and that levels are negatively correlated with BMI [50]. The association between vitamin D deficiency and MetS were also confirmed by Cao et al. [51]. Since vitamin D deficiency is associated with the risk of many degenerative diseases, low vitamin D levels are also associated with accelerated biological aging [52].

The negative correlation between vitamin D and NLRP3 in the non-MetS group may be due to its anti-inflammatory effect. Rao et al. and Tunbridge et al. documented that the vitamin D receptor inhibited NLRP activation [53,54]. Positive correlations between vitamin D, HDL, and GDF11 have shown the protective effect of vitamin D in relation to, e.g., cardiovascular or neurodegenerative diseases [55]. The positive correlation between vitamin D and AGEs in the non-MetS group is surprising. The results of various studies suggest that vitamin D tends to reduce AGEs [56,57].

The expression of GDF15 reflected cellular and mitochondrial stress, which increases with aging and the presence of chronic inflammatory diseases, including metabolic and cardiovascular disorders [29,58].

In our study, GDF15 production was enhanced in the MetS group and the 35–50 MetS subgroup. In their study, Carballo-Casla et al. enrolled 1938 persons aged 65 years or older (1471 non-MetS and 521 MetS). GDF15 levels were higher in MetS persons and were associated with high waist circumference, increased glucose levels, and HDL [59] In our study, GDF15, as expected, correlated with age, but only in the MetS group, and correlated in non-MetS individual with total cholesterol, nonHDL, and LDL; these parameters are associated with the risk of cardiometabolic disorders and inflammation. GDF15 increases due to aging and inflammation, and physical activity can reduce its production [60].

DNA/RNA damage was higher in MetS compared to non-MetS individuals, without statistical differences in the subgroups. MetS is accompanied by increased ROS production and inflammation [61,62]. Bhutia et al. achieved the same results as we did. Oxidative DNA damage is associated with the formation of DNA breaks. A higher number of DNA breaks have been observed in MetS. Demirbag et al. showed that MetS is associated with oxidative stress, a decline in antioxidant capacity, and increases in DNA damage [63]. Using the micronucleus test and comet assay, Karaman et al. demonstrated that MetS is associated with DNA strand breaks [64]. It is well documented that oxidative stress and DNA damage is also associated with the acceleration of degenerative processes, carcinogenesis, and aging [65].

We cannot explain the negative correlation between NLRP3 and DNA/RNA damage in non-MetS. Oxidative stress induces DNA/RNA damage and the activation of NLRP3. Higher levels of vitamin D reduce both NLRP3 activation and DNA-damaging oxidative stress. On the other hand, we also observed a positive correlation between vitamin D and AGEs in non-MetS, which can induce oxidative stress as well as inflammation. The question is whether vitamin D acts more as an anti-inflammatory or antioxidant factor in non-MetS persons [66,67]. The levels of DNA/RNA damage also correlated with age and negatively with GDF11 in non-MetS and positively with sirtuin 1 in both groups. We have already written above that aging is associated with increased oxidative DNA/RNA damage and GDF11 acts as a rejuvenating factor. Thus, its decline may be associated with higher DNA/RNA damage. The positive correlation between DNA/RNA damage and sirtuin 1 can be a compensatory mechanism. Sirtuin 1 is involved in DNA reparation [14].

We did not detect differences in the levels of AGEs, sirtuin 1, GDF11 (slightly higher in MetS), NAD^+^, klotho (slightly lower in MetS), and NLRP3. Although there are no significant differences, there is a pattern that suggests that persons with MetS may biologically age at faster rates. 

Our study showed that the presence of MetS is associated with an increase in various markers of aging. Subgroup analysis according to age showed that the differences of some parameters depended on age. This may indicate that the processes that occur in the body during MetS are damaging, inducing not only inflammation and oxidative stress but also accelerating aging. 

## 5. Conclusions

The results of our study show that the presence of MetS alters the levels of selected markers of aging. This suggests that biological age may be older than chronological age in individuals with metabolic syndrome compared to healthy controls. This increases the risk of developing other chronic diseases, including cardiovascular, metabolic, inflammatory, neurodegenerative or cancer diseases, and the risk of premature death from all causes, especially in younger people, where differences were found between some parameters, while no differences were identified in persons over 50 years of age. Thus, some parameters depend not only on the presence of MetS but also on age. These data also show that metabolic syndrome, the prevalence of which is increasing in the population worldwide, is a serious disease and prevention and comorbidities need to be targeted.

Knowledge of biological age can contribute to the early recognition of other health risks, the initiation of treatment to prevent complications, and death. It can also help to decide, when initiating therapy for associated diseases (e.g., cancer), whether it is appropriate and manageable for the patient.

## 6. Study Limitations

The limitations of this study mainly concern the number of participants when divided into subgroups according to age. It should also be kept in mind that although selected markers are associated with aging, their levels may also reflect the presence of other pathological conditions. However, these may arise from aging or accelerate aging. Another limitation of this study may be the inability to determine the influence of genetics or lifestyle on the selected factors. These data are not available. However, with regard to lifestyle, it can be said that people with metabolic syndrome often do not follow healthy lifestyle rules, as evidenced by higher lipid levels, blood pressure, and BMI compared to people without metabolic syndrome. The homogeneity of a White population in the Czech Republic is a limitation of the study when extrapolating the results worldwide.

## Figures and Tables

**Table 1:** Levels of biochemical markers and parameters associated with MetS in non-MetS and MetS participants.

	N	Median	Q1	Q3	Min.	Max.	*p* Value
BMI (body mass index)							
Non-MetS	111	24.91	22.74	27.12	18.21	32.53	*p* < 0.001
Mets	58	29.65	27.44	32.81	22.59	45.17	
Systolic blood pressure mmHg							
Non-MetS	111	122	112	131	100	151	*p* < 0.001
MetS	58	136	130	143	108	163	
Diastolic blood pressure mmHg							
Non-MetS	111	78	71	84.5	60	99	*p* < 0.001
Mets	58	88	85	95.0	12	100	
Waist cm							
Non-MetS	111	80.0	75	90	54	109	*p* < 0.001
Mets	58	103.5	94	110	75	125	
Age years							
Non-MetS	111	38.00	29.59	51.57	19.62	65.92	*p* < 0.01
Mets	58	45.89	40.10	53.98	22.43	63.54	
Total cholesterol mmol/L							
Non-MetS	111	4.61	4.21	5.07	2.39	7.16	*p* < 0.01
Mets	58	5.02	4.65	5.75	2.90	6.91	
LDL (low density lipoprotein) mmol/L							
Non-MetS	111	2.58	2.05	3.03	0.85	4.60	*p* < 0.01
Mets	58	3.05	2.66	3.75	1.37	4.52	
HDL (high density lipoprotein) mmol/L							
Non-MetS	111	1.27	1.46	1.80	0.75	2.63	*p* < 0.001
Mets	58	0.96	1.08	1.25	0.72	2.03	
nonHDL (total non-high density lipoprotein) mmol/L							
Non-MetS	111	3.13	2.52	3.58	1.52	5.50	*p* < 0.001
Mets	58	3.92	3.52	4.65	1.60	5.88	
Triglycerides mmol/L							
Non-MetS	111	1.07	0.78	1.41	0.39	3.04	*p* < 0.001
Mets	58	1.92	1.52	2.50	0.51	11.05	
Fasting glucose mmol/L							
Non-MetS	111	4.53	3.99	4.90	2.37	7.52	*p* < 0.01
Mets	58	4.86	4.36	5.33	3.76	7.81	

Legend: Q1 and Q3, first and third quartiles; N, number of participants; Min and Max, minimal and maximal values.

**Table 2:** Significant differences in levels of markers of aging in non-MetS and MetS participants divided into groups according to age.

	N	Median	Q1	Q3	Min	Max	
Group > 35							
Follistatin pg/mL							
Non-MetS	48	1045.45	849.70	1840.28	322.21	5976.39	*p* < 0.05
MetS	9	888.11	635.54	943.13	628.50	1082.47	
Vitamin D ng/mL							
Non-MetS	48	23.19	20.35	28.11	14.11	37.31	*p* < 0.05
MetS	9	18.61	17.47	21.43	11.84	28.64	
Group 35–50							
Follistatin pg/mL							
Non-MetS	31	1100.24	837.97	1261.45	477.58	2190.54	*p* < 0.01
MetS	28	1316.68	1094.69	1723.55	863.00	19,006.93	
GDF15 pg/mL							
Non-MetS	31	255.75	231.20	333.97	117.71	412.30	*p* < 0.05
MetS	28	305.99	262.17	350.59	167.02	460.99	
Group > 50	Without significant differences						

Legend: Q1 and Q3, first and third quartiles; N, number of participants; Min and Max, minimal and maximal values.

**Table 3:** Statistically significant correlations between parameters in MetS and non-MetS subjects.

MetS	Spearman’s Rho	*p* Value	Non-MetS	Spearman’s Rho	*p* Value
NLRP3					
Age	xx	xx		0.212	0.026
GDF11	xx	xx		0.320	0.0007
DNA/RNA	−0.3506	0.007		−0.348	0.0002
Vitamin D	xx	xx		−0.302	0.001
Follistatin	0.288	0.028		xx	xx
Telomerase	xx	xx		−0.248	0.009
Klotho	xx	xx		0.207	0.029
Klotho					
Age	xx	xx		0.232	0.015
HDL	xx	xx		0.259	0.006
Sirtuin 1	xx	xx		−0.237	0.013
NAD	0.293	0.026		xx	xx
GDF11	0.297	0.025		0.302	0.002
Telomerase	xx	xx		−0.207	0.029
Telomerase					
Fasting glucose	−0.319	0.015		−0.1926	0.04297
Sirtuin 1	0.637	1.011 × 10^−7^		0.6809	5.151 × 10^−16^
NAD	xx	xx		−0.2940	0.00179
GDF11	−0.261	0.050		−0.3934	2.537 × 10^−5^
DNA/RNA	0.369	0.004		0.3881	2.565 × 10^−5^
AGEs	0.372	0.004		0.4035	1.344 × 10^−5^
Follistatin					
Age	0.332	0.011		xx	xx
Cholesterol	xx	xx		0.2269	0.0166
nonHDL	xx	xx		0.1986	0.0367
TAG	xx	xx		0.2210	0.0198
GDF15	0.4412	0.0006		0.3303	0.0004
AGE	0.4073	0.0015		xx	xx
AGE					
nonHDL	xx	xx		0.2681	0.0044
LDL	xx	xx		0.2778	0.0032
Sirtuin 1	0.2770	0.0370		0.3661	9.760 × 10^−5^
GDF11	xx	xx		−0.3699	8.159 × 10^−5^
GDF15	0.31757	0.0151		xx	xx
Vitamin D	xxx	xx		0.3040	0.0012
Vitamin D					
BMI	−0.3553	0.0062		−0.2072	0.029
waist	−0.3231	0.0134		xx	xx
HDLC	xx	xx		0.2732	0.0037
GDF11	xx	xx		−0.3169	0.0008
DNA/RNA damage					
BMI	xx	xx		0.2178	0.0217
Sirtuin 1	0.3206	0.0150		0.2947	0.0020
GDF11	xx	xx		−0.2793	0.0034
GDF15					
Age	0.6324	1.008 × 10^−7^		0.4913	4.378 × 10^−8^
Cholesterol	xx	xx		0.3764	4.660 × 10^−5^
nonHDL	xx	xx		0.3520	0.0002
LDL	xx	xx		0.3467	0.0002
GDF11					
Sirtuin 1	−0.4191	0.0012		−0.49291	5.991 × 10^−8^
NAD	0.4409	0.0006		0.2543	0.0079
NAD					
HDL	0.2808	0.0328		xx	xx
Sirtuin 1	−0.3378	0.01038		−0.1907	0.0480

Legend: xx, without significant correlation.

**Table 4:** Differences between correlations in non-MetS participants.

M-	NLRP3	Klotho	Telom	Folli	AGE	Vit D	DNA	GDF15	GDF11	NAD	Sirt 1
age	*	*						***			*
BMI						*	*				
waist											
GLU			*								
CHOL				*				***			
HDL		**				**					
nHDL				*	**			***			
TAG				*							
LDL					**			***			
Sirt 1		*	***		***		**		***	*	x
NAD			**						**	x	
GDF11	***	**	***		***	***	**		x		
GDF15				***				x			
DNA	***		***				x				
Vit D	**				**	x					
AGE			***		x						
Folli				x							
Telom	**	*	x								
Klotho	*	x									

Legend: GLU, fasting glucose; CHOL, total cholesterol; HDL, high density lipoprotein; nHDL, non-high-density lipoprotein; TAG, triglycerides; Vit D, vitamin D; Telom, telomerase; Folli, follistatin; Sirt 1, sirtuin 1; DNA, DNA/RNA damage; *, *p* < 0.05; **, *p* < 0.01; ***, *p* < 0.001; x, not applicable.

**Table 5:** Differences between correlations in MetS participants.

MetS	NLRP3	Klotho	Telom	Folli	AGE	Vit D	DNA	GDF15	GDF11	NAD	Sirt 1
age				*				***			
BMI						**					
waist						*					
GLU			*								
CHOL											
HDL										*	
nHDL											
TAG											
LDL											
Sirt 1			***		*		*		**	*	x
NAD		*							***	x	
GDF11		*	*						x		
GDF15				***	*			x			
DNA	**		**				x				
Vit D						x					
AGE			**	**	x						
Follistatin	*			x							
Telomerase			x								
Klotho		x									

Legend: GLU, fasting glucose; CHOL, total cholesterol; HDL, high density lipoprotein; nHDL non-high-density lipoprotein; TAG, triglycerides; Vit D, vitamin D; Telom, telomerase; Folli, follistatin; Sirt 1, sirtuin 1; DNA, DNA/RNA damage; *, *p* < 0.05; **, *p* < 0.01; ***, *p* < 0.001; x, not applicable.

## Data Availability

The data supporting published results are available from the corresponding author if requested.

## Supplementary Materials

The following supporting information can be downloaded at: https://www.mdpi.com/article/10.3390/biomedicines12010242/s1, Table S1: Metabolic syndrome data according to age, gender.
