# Supplementary material for: The Influence of Metabolic Syndrome on Potential Aging Biomarkers in Participants with Metabolic Syndrome Compared to Healthy Controls
Source: Biomedicines. 2024 Jan 22;12(1):242. doi: 10.3390/biomedicines12010242 (PMC10813522; doi:10.3390/biomedicines12010242)
Supplement: Supplementary file 1 [file biomedicines-12-00242-s001.zip › biomedicines-2725778-supplementary.pdf]

Supplementary Table S1. Metabolic syndrome data according to age, gender.

**Group <35**							
	**N**	**25q**	**Median**	**75q**	**Min**	**Max**	
BMI no MS	48	22.56	24.56	27.49	18.21	32.53	$p<0.01$
BMI yes MS	9	27.13	28.48	31.07	25.31	45.17	
	**N**	**25q**	**Median**	**75q**	**Min**	**Max**	
sys no MS	48	109.75	121	131	100	151	NS
sys yes MS	9	122.00	130	143	108	150	
	**N**	**25q**	**Median**	**75q**	**Min**	**Max**	
dia no MS	48	70	75.5	82.25	60	95	$p<0.01$
dia yes MS	9	85	85.0	90.00	81	90	
	**N**	**25q**	**Median**	**75q**	**Min**	**Max**	
waist no MS	48	73	78.5	88.5	66	109	$p<0.001$
waist yes MS	9	94	99.0	110.0	88	116	
	**N**	**25q**	**Median**	**75q**	**Min**	**Max**	
GLU no MS	48	3.97	4.50	4.85	2.42	5.93	NS
GLU yes MS	9	4.27	4.62	4.79	3.96	4.87	
	**N**	**25q**	**Median**	**75q**	**Min**	**Max**	
CHOL no MS	48	3.93	4.29	4.63	2.39	6.77	NS
CHOL yes MS	9	3.81	4.35	4.88	2.90	6.12	
	**N**	**25q**	**Median**	**75q**	**Min**	**Max**	
nonHDL no MS	48	2.28	2.65	3.21	1.52	5.50	NS
nonHDL yes MS	9	03.07	3.58	3.94	1.60	4.87	
	**N**	**25q**	**Median**	**75q**	**Min**	**Max**	
TAG no MS	48	0.8	1.14	1.39	0.47	2.00	$p<0.05$
TAG yes MS	9	1.2	1.87	2.33	0.51	3.14	
	**N**	**25q**	**Median**	**75q**	**Min**	**Max**	
HDLC no MS	48	1.33	1.46	1.72	0.87	2.25	$p<0.001$
HDLC yes MS	9	0.88	0.94	01.02	0.74	1.30	
	**N**	**25q**	**Median**	**75q**	**Min**	**Max**	
LDLV no MS	48	1.94	2.13	2.66	0.85	4.6	NS
LDLV yes MS	9	2.12	2.54	3.43	1.37	4.1	
**Group 30–50**							
	**N**	**25q**	**Median**	**75q**	**Min**	**Max**	
BMI no MS	31	24.12	25.62	27.79	19.36	32.00	$p<0.001$

BMI yes MS	28	26.88	29.58	32.98	23.15	42.45	
	**N**	**25q**	**Median**	**75q**	**Min**	**Max**	
sys no MS	31	112.5	120.0	130	100	148	p<0.001
sys yes MS	28	130.0	136.5	141	112	160	
	**N**	**25q**	**Median**	**75q**	**Min**	**Max**	
dia no MS	31	72.5	78.0	84.5	64	91	p<0.001
dia yes MS	28	82.5	89.5	96.0	12	100	
	**N**	**25q**	**Median**	**75q**	**Min**	**Max**	
waist no MS	31	78	85	88.00	54	102	p<0.001
waist yes MS	28	91	100	107.75	75	125	
	**N**	**25q**	**Median**	**75q**	**Min**	**Max**	
GLU no MS	31	4.20	4.55	4.86	3.52	6.61	p<0.05
GLU yes MS	28	4.49	4.94	5.43	3.76	7.81	
	**N**	**25q**	**Median**	**75q**	**Min**	**Max**	
CHOL no MS	31	4.42	4.73	05.06	3.44	6.12	NS
CHOL yes MS	28	4.63	05.06	5.66	3.64	6.88	
	**N**	**25q**	**Median**	**75q**	**Min**	**Max**	
nonHDL no MS	31	2.62	3.25	3.88	1.79	5.29	p<0.05
nonHDL yes MS	28	3.27	3.96	4.60	2.51	5.88	
	**N**	**25q**	**Median**	**75q**	**Min**	**Max**	
TAG no MS	31	0.80	01.04	1.56	0.39	03.04	p<0.001
TAG yes MS	28	1.65	2.10	2.53	0.92	11.05	
	**N**	**25q**	**Median**	**75q**	**Min**	**Max**	
HDLC no MS	31	1.21	1.40	1.57	0.75	2.63	p<0.001
HDLC yes MS	28	0.96	01.06	1.13	0.75	1.55	
	**N**	**25q**	**Median**	**75q**	**Min**	**Max**	
LDLV no MS	31	2.37	2.73	3.16	1.41	4.55	NS
LDLV yes MS	27	2.60	2.93	3.43	1.64	4.52	
**Group > 50**							
	**N**	**25q**	**Median**	**75q**	**Min**	**Max**	
sys no MS	32	113.5	124.5	134.25	100	150	p<0.001
sys yes MS	21	130.0	136.0	145.00	119	163	
	**N**	**25q**	**Median**	**75q**	**Min**	**Max**	
dia no MS	32	74.75	80	85.25	63	99	p<0.001
dia yes MS	21	85.00	87	90.00	56	100	

	N	25q	Median	75q	Min	Max	
waist no MS	32	75	78.5	91.25	68	99	p<0.001
waist yes MS	21	103	106.0	112.00	84	122	
	**N**	**25q**	**Median**	**75q**	**Min**	**Max**	
GLU no MS	32	3.71	4.58	5.10	2.37	7.52	NS
GLU yes MS	21	4.49	05.05	5.51	3.76	6.16	
	**N**	**25q**	**Median**	**75q**	**Min**	**Max**	
CHOL no MS	32	4.59	5.15	5.74	4.15	7.16	NS
CHOL yes MS	21	4.91	5.46	06.04	4.41	6.91	
	**N**	**25q**	**Median**	**75q**	**Min**	**Max**	
nonHDL no MS	32	2.95	3.44	4.25	02.06	5.17	p<0.001
nonHDL yes MS	21	3.76	4.37	4.75	3.34	5.47	
	**N**	**25q**	**Median**	**75q**	**Min**	**Max**	
TAG no MS	32	0.78	01.07	1.31	0.60	02.08	p<0.001
TAG yes MS	21	1.38	1.82	2.46	01.04	3.32	
	**N**	**25q**	**Median**	**75q**	**Min**	**Max**	
LDLV no MS	32	2.56	2.95	3.47	1.75	4.48	p<0.05
LDLV yes MS	21	3.00	3.23	3.79	2.68	4.52	
	**N**	**25q**	**Median**	**75q**	**Min**	**Max**	
HDLC no MS	32	1.27	1.79	02.04	1.00	2.54	p<0.001
HDLC yes MS	21	01.01	1.22	1.33	0.72	02.03	
**Gender**							
	**N**	**25q**	**Median**	**75q**	**Min**	**Max**	
BMI Female	84	22.66	24.96	28.55	18.21	45.17	p<0.01
BMI Male	85	25.28	27.16	29.36	18.79	38.06	
	**N**	**25q**	**Median**	**75q**	**Min**	**Max**	
sys Female	84	110.75	120	130	100	163	p<0.001
sys Male	85	123.00	133	141	100	162	
	**N**	**25q**	**Median**	**75q**	**Min**	**Max**	
dia Female	84	71.75	79.5	87	60	100	p<0.05
dia Male	85	77.00	84.0	90	12	100	
	**N**	**25q**	**Median**	**75q**	**Min**	**Max**	
waist Female	84	73	78	88.25	66	125	p<0.001
waist Male	85	88	95	104.00	54	122	
	**N**	**25q**	**Median**	**75q**	**Min**	**Max**	

GLU Female	84	4.00	4.62	5.20	2.76	7.81	NS
GLU Male	85	4.12	4.63	4.97	2.37	6.46	
	**N**	**25q**	**Median**	**75q**	**Min**	**Max**	
CHOL Female	84	4.22	4.62	5.30	2.90	7.16	NS
CHOL Male	85	4.42	4.81	5.36	2.39	6.91	
	**N**	**25q**	**Median**	**75q**	**Min**	**Max**	
nonHDL Female	84	2.48	03.06	3.66	1.52	5.77	p<0.001
nonHDL Male	85	3.17	3.63	4.35	1.52	5.88	
	**N**	**25q**	**Median**	**75q**	**Min**	**Max**	
TAG Female	84	0.78	1.1	1.50	0.39	4.48	p<0.001
TAG Male	85	01.08	1.5	02.04	0.47	11.05	
	**N**	**25q**	**Median**	**75q**	**Min**	**Max**	
HDLC Female	84	1.31	1.57	1.90	0.87	2.63	p<0.001
HDLC Male	85	1.00	1.17	1.36	0.72	2.21	
	**N**	**25q**	**Median**	**75q**	**Min**	**Max**	
LDLV Female	84	02.01	2.52	3.14	1.16	4.52	p<0.01
LDLV Male	84	2.56	2.92	3.32	0.85	4.60	

Legend: N, number of participants; NS, non-significant p value; Yes MS, participants with metabolic syndrome; No MS, participants without metabolic syndrome; Sys: systolic blood pressure; Dia: diastolic blood pressure; Waist in cm; GLU: fasting glucose; CHOL: total cholesterol; nonHDL: non high density lipoproteins; TAG: triacylglycerols; HDLC: high density lipoproteins; LDLV: low density lipoproteins
